# A Lithuanian Case of Tyrosinemia Type 1 with a Literature Review: A Rare Cause of Acute Liver Failure in Childhood

**DOI:** 10.3390/medicina60010135

**Published:** 2024-01-11

**Authors:** Rūta Rokaitė, Agnė Čibirkaitė, Vykinta Zeleckytė, Gabija Lazdinytė, Mindaugas Dženkaitis

**Affiliations:** 1 Department of Pediatrics, Medical Academy, Lithuanian University of Health Sciences, LT 44307 Kaunas, Lithuania; 2 Faculty of Medicine, Medical Academy, Lithuanian University of Health Sciences, LT 44307 Kaunas, Lithuania; 3 School of Medicine, College of Health and Agricultural Sciences, University College Dublin, Belfield, D04 V1W8 Dublin, Ireland

**Keywords:** tyrosinemia type 1, acute liver failure, children, nitisinone (NTBC)

## Abstract

Hereditary type 1 tyrosinemia (HT1) is a rare inherited autosomal recessive disorder of tyrosine metabolism, characterized by progressive liver damage, dysfunction of kidney tubules, and neurological crises. In the course of this disease, due to the deficiency of the enzyme fumarylacetoacetate hydrolase (FAH), toxic intermediate metabolites of tyrosine breakdown, such as fumarylacetoacetate (FAA), succinylacetoacetate (SAA), and succinylacetone (SA), accumulate in liver and kidney cells, causing cellular damage. Because of this, an increased SA concentration in the blood or urine is pathognomonic of HT1. In the year 2000, HT1 was diagnosed in Lithuania for the first time, and this was the first time when a specific treatment for HT1 was administered in the country. Over two decades, four cases of this disease have been diagnosed in Lithuania. In the first of these patients, the disease was diagnosed in infancy, manifesting as liver damage with liver failure. Treatment with nitisinone was initiated, which continues to be administered, maintaining normal liver function. Liver transplantation was performed on two subsequent patients due to complications of HT1. It is crucial to diagnose HT1 as early as possible in order to reduce or completely eliminate complications related to the disease, including progressive liver failure and kidney dysfunction, among others. This can only be achieved by conducting a universal newborn screening for tyrosinemia and by starting treatment with nitisinone (NTBC) before the age of 1 month in all cases of HT1. However, in those countries where this screening is not being carried out, physicians must be aware of and consider this highly rare disorder. They should be vigilant, paying attention to even minimal changes in a few specific laboratory test results—such as unexplained anemia alongside neutropenia and thrombocytopenia—and should conduct more detailed examinations to determine the causes of these changes. In this article, we present the latest clinical case of HT1 in Lithuania, diagnosed at the Children’s Diseases’ Clinic of the Lithuanian University of Health Sciences (LUHS) Hospital Kaunas Clinics. The case manifested as life-threatening acute liver failure in early childhood. This article explores and discusses the peculiarities of diagnosing this condition in the absence of universal newborn screening for tyrosinemia in the country, as well as the course, treatment, and ongoing monitoring of patients with this disorder.

## 1. Introduction

Hereditary type 1 tyrosinemia (HT1) is a rare inherited autosomal recessive disorder of tyrosine metabolism, characterized by progressive liver damage, dysfunction of kidney tubules, and neurological crises, with a long-term risk of hepatocellular carcinoma (HCC) [1,2,3]. It is considered to be the most severe disorder in tyrosine metabolism [3]. The amino acid tyrosine enters the body with food and is also formed in the body from phenylalanine. The breakdown of tyrosine mainly occurs in the cytosol of liver cells and involves five enzymes. In the final step of this process, the enzyme fumarylacetoacetate hydrolase (FAH) breaks down fumarylacetoacetate (FAA) into fumarate and acetoacetate. HT1 is a disorder that is characterized by an inherited deficiency of FAH, leading to the accumulation of toxic intermediate metabolites of tyrosine breakdown—such as FAA, SAA (succinylacetoactetate), and SA (succinylacetone)—in liver and kidney cells, causing cellular damage [3].

The gene encoding FAH is located on chromosome 15q23-q25 and consists of 14 exons [4]. About 100 different mutations responsible for HT1 are known [5]. The most common mutations specific to certain ethnic groups have been identified. In the Canadian province of Quebec, the c.1062+5G>A (IVS12+5G>A) mutation is prevalent in 86% of cases [6]. The same mutation is common in Northern Europe, constituting 45% of the alleles responsible for HT1. In addition, the p.W262X mutation is found in 80% of cases in Finland, while the c.554-1G>T (IVS6-1G>T) mutation accounts for 64% of pathogenic alleles in Southern Europe [3].

The global prevalence of HT1 is approximately 1 in 100,000–120,000 live births [7]. The frequency of HT1 is higher in two specific regions of the world. The Nordic countries of Norway and Finland constitute the first region with a higher HT1 prevalence, with frequencies of 1 in 74,800 and 1 in 60,000 live births, respectively [8,9]. The second of these regions is the Canadian province of Quebec, which has an HT1 frequency of 1 in 16,000 [10].

Due to its rarity, HT1 is not that well-known and may go unrecognized if universal newborn screening is not being performed. In those countries where universal screening for tyrosinemia is conducted, HT1 is diagnosed within the first month of life. However, in such countries as Lithuania where universal screening for this condition is not being performed, the disease is only diagnosed when serious clinical symptoms occur, significantly affecting patient outcomes.

In Lithuania, HT1 was diagnosed and nitisinone treatment was administered for the first time in the year 2000. Over a period of two decades, four cases of this disease have been diagnosed in Lithuania. In the first of these cases, the disease was diagnosed in infancy, manifesting as liver damage with liver failure. Treatment with nitisinone was initiated, and it is still currently ongoing, maintaining the patient’s normal liver function. Subsequently, two other patients were diagnosed with HT1 in childhood (at the age of 3–4 years), developing liver cirrhosis and hepatocellular carcinoma, which led to liver transplantations in both cases. After the liver transplantation was performed, these patients did not receive any specific HT1 treatment and reported feeling well.

In this article, we present the fourth and latest clinical case of HT1 in Lithuania, diagnosed at the Children’s Diseases’ Clinic of the Lithuanian University of Health Sciences (LUHS) Hospital Kaunas Clinics.

## 2. Case Report

The girl was born from a first pregnancy and delivery. She was born prematurely at 34 weeks of gestation, weighing 2600 g, delivered via natural birth without hypoxia (Apgar score 10–10) and without any visible abnormalities. The mother’s pregnancy was uncomplicated, and the newborn period of the patient proceeded smoothly. There are no known hereditary diseases in the family.

The patient was breastfed from birth, and complementary foods were introduced according to her age. At first, the patient’s growth and development were normal. From the age of one and a half years, the patient began to suffer from frequent viral upper respiratory tract infections. A general blood test revealed mild normocytic normochromic anemia (Hb 98 g/L), neutropenia (0.7 × 10^9^/L), and thrombocytopenia (117 × 10^9^/L). Due to the observed anemia, various iron preparations were prescribed by the family physician, which the patient took continuously for 5 months. However, with no improvement and persistent mild normocytic normochromic anemia, the patient, who was 2 years old at the time, was referred to the Children’s Diseases’ Clinic at the LUHS Hospital Kaunas Clinics for further examination.

Upon examination, the patient had an enlarged, yet painless, abdomen, palpable liver about 3 cm below the right costal margin, and an enlarged spleen. Jaundice was not observed. The patient’s physical development was normal for her age.

After inpatient laboratory tests were conducted, the complete blood count showed mild normocytic normochromic anemia (Hb 103 g/L), and other laboratory tests indicated acute liver failure (significantly reduced prothrombin complex (SPA) activity, hyperammonemia, and mild hypoalbuminemia) with marked liver cholestasis (marked elevation of gamma-glutamyltransferase (GGT) and alkaline phosphatase (ALP)). The kidney function indicators were normal (Table 1).

**Figure 1:**
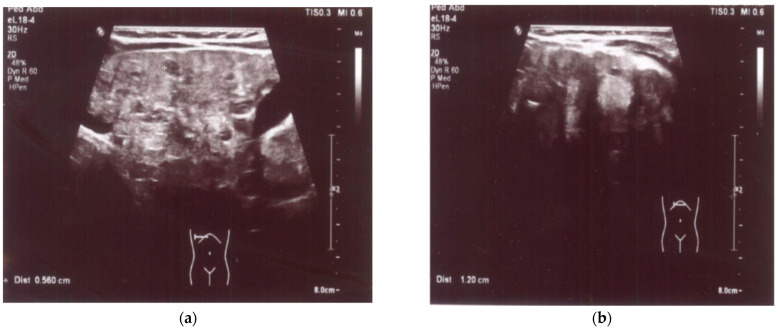
(**a**) US: liver with multiple smaller hypoechoic lesions. (**b**) US: hypoechoic lesion up to 1.2 cm in size in the left hepatic lobe.

An enlarged liver (cranio-caudal dimension about 9.5–10 cm) with an irregular contour and diffusely heterogeneous parenchyma was observed both in ultrasound and in abdominal computed tomography (CT) imaging studies. Multiple smaller hypoechoic lesions were noted on abdominal ultrasonography (US) (Figure 1a,b), while hypodense lesions up to ~0.5 cm in size, with a larger lesion in the left lobe up to 1.2–1.6 cm in size, were also observed at the same locations on abdominal CT. Additionally, the spleen was enlarged and homogenous, measuring about 10 cm by 4 cm in size. A liver biopsy revealed signs of cirrhosis with steatosis. No evidence of neoplastic changes was found.

While investigations were being conducted to determine the cause of the liver damage, the patient’s condition rapidly deteriorated over the course of a week. She developed fever, became lethargic, jaundice became prominent, and the abdominal distension gradually increased. Laboratory tests showed a negative trend (Table 1), and signs of ascites appeared on ultrasound. On the fifth day of hospitalization, the patient was diagnosed with sepsis (blood cultures showed *S. aureus* growth, sensitive to cefazolin), which further aggravated the patient’s clinical condition. By administering antibiotics along with continued symptomatic treatment and addressing the worsening anemia by performing a red blood cell mass transfusion, the patient’s condition was somewhat stabilized.

To elucidate the etiology of the liver damage, various tests were conducted, but there were no data to support viral nor autoimmune hepatitis, nor alpha-1 antitrypsin deficiency. However, given a significantly elevated alpha-fetoprotein (AFP) concentration (47,863 kU/L) and concurrent acute liver failure with marked liver cholestasis, tyrosinemia was suspected.

A blood plasma amino acid analysis revealed a fourfold increase in tyrosine concentration (482.51 µmol/L, normal range 22.43–125.11), while an analysis of organic acids in urine indicated significantly elevated succinylacetone (10,833.18 µmol/mmol creatinine, normal <2.67), 3-phenyllactic acid (91.60 µmol/mmol creatinine, normal <2.0), and P-hydroxyphenyllactic acid (481.29 µmol/mmol creatinine, normal <27.06). The results of the blood amino acid and urine organic acid analyses were characteristic of tyrosinemia. Subsequently, a diagnosis of HT1 was confirmed via molecular genetic testing. A pathogenic homozygous variant 000137.4(FAH):c.[1062+5G>A];[1062+5G>A] was identified in the FAH gene. Consanguinity was not ruled out due to an incomplete family history regarding one of the parents.

Upon suspecting HT1, treatment with nitisinone (1 mg/kg/day) was initiated. At the time, the patient was 2 years old. After the first day of treatment, a positive clinical effect was already observed—the patient’s overall condition started to improve. The patient became more active, her fever subsided, and positive dynamics were noted in the laboratory test results (Table 1).

**Figure 2:**
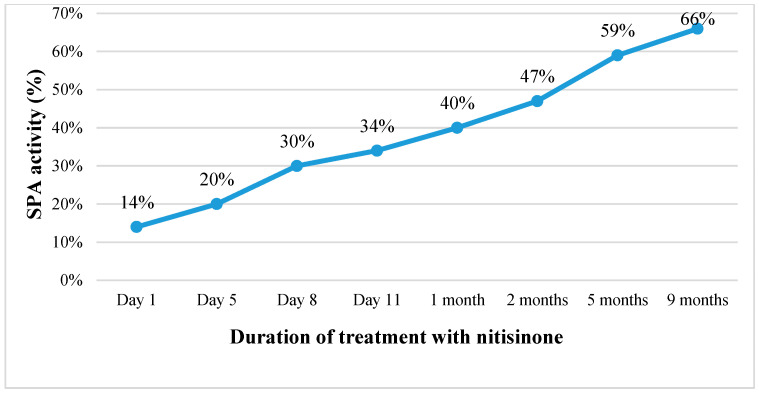
Changes in SPA activity (%) with the duration of treatment with nitisinone. (SPA is a measure of liver function, with lower values indicating impaired hepatic functioning).

Continuing the treatment with nitisinone, the patient’s condition gradually improved. Within one and a half weeks, the ascites disappeared, the abdominal circumference was reduced by about 6 cm, and the laboratory tests indicated improving liver function (SPA increased from 14% to 66%, albumin levels rose from 26 to 40.1 g/L) with decreasing AFP concentration in the blood (from 47,863.6 kU/L before commencing treatment to 100.2 kU/L after nine months of treatment) (Table 1; Figure 2). However, after the first month of treatment, an increasing level of GGT in the blood was observed. Therefore, ursodeoxycholic acid (UDCA) was also prescribed, which had a positive effect in reducing cholestasis in the small bile ducts. Within the course of treatment, the ultrasound profile of the liver and its size remained unchanged (~9.5 cm in the cranio-caudal dimension), while there was a slight decrease in the size of the spleen (to 9.0 cm by 2.5 cm in size).

During the course of treatment, blood tyrosine levels fluctuated between 400 and 520 µmol/L, while the level of succinylacetone (SA) in urine gradually decreased from 8.55 to 4.14 µmol/mmol creatinine. This was achieved by administering nitisinone (1 mg/kg/day) and maintaining a protein intake of 1–2 g/kg/day through the diet, while restricting the intake of foods rich in tyrosine and phenylalanine.

## 3. Discussion

According to the literature, HT1 can manifest at any age from infancy to adulthood, and it is characterized by a wide spectrum of clinical symptoms. The severity of the disease is generally negatively correlated with the age at which the disease manifests. HT1 is classified into three types based on the level of liver function impairment: acute, subacute, and chronic, although clear boundaries between them are not defined. The acute type is considered the most common and severe form of HT1. It typically begins a few weeks after birth with hepatomegaly, poor development, diarrhea, vomiting, jaundice, and, in severe cases, it progresses to liver damage, leading to liver failure [3]. Sepsis often develops, and due to tubular kidney dysfunction, signs of hypophosphatemic rickets may appear early on in the course of the disease. When the disease manifests in infancy or childhood (from a few months to approximately 1–2 years after birth), HT1 may present as the subacute type—with impaired liver function, signs of rickets, or less specific symptoms, such as a tendency to bleed, growth impairment, hepatomegaly, and hepatosplenomegaly. In contrast, chronic-type liver function impairment progresses slowly but can ultimately lead to cirrhosis or liver failure. It may also result in mild growth retardation and subclinical rickets. Bone marrow function may be compromised in some HT1 patients, leading to hematologic abnormalities, such as thrombocytopenia, leukopenia, and anemia [10].

Our article describes a case of the subacute type of HT1, as the initial symptoms of the disease in our patient could be observed at the age of one and a half years when anemia, neutropenia, and thrombocytopenia of unknown origin were accidentally detected. However, at that time, no examinations assessing the patient’s liver condition were performed. The patient was only diagnosed with HT1 at the age of two years when she was found to have liver damage with progressive liver failure and hepatosplenomegaly, but no signs of kidney dysfunction or rickets were present.

Having a functional universal newborn screening program for tyrosinemia is crucial for diagnosing HT1 early. In countries where testing for tyrosinemia is part of routine newborn screening, HT1 is usually identified before the onset of symptoms, often by the age of one month [10,11,12]. However, in many countries around the world, including in Lithuania where this condition is not included in the newborn screening program, HT1 is often diagnosed only when clinical signs of liver damage or symptoms of liver failure or kidney dysfunction become apparent.

Various degrees of liver cell damage and biochemical changes indicative of liver synthetic dysfunction can be identified in patients with HT1. Significant alterations in coagulation indicators are observed not only in acute cases, but also quite frequently in patients with the chronic form of the disease. Coagulopathy is an early sign, manifesting even in the absence of other clinical signs of impaired liver function [3]. When assessing liver enzyme changes, HT1 is characterized by elevated GGT and/or ALP levels, while the levels other liver enzymes may remain normal or only mildly elevated. The bilirubin concentration in the serum can also be normal or only slightly increased, as in the case of our patient.

Patients with the acute form of the disease often exhibit a significantly elevated AFP concentration in the blood, although AFP levels could be within normal range in those with the chronic form. A heightened AFP level alone is not a diagnosis of HT1; however, conversely, an increased SA level in the blood or urine is pathognomonic of HT1 [1,2,10,13]. While an elevated AFP level alone is not an HT1 diagnosis, the presence of elevated liver enzymes such as GGT and ALP (together with elevated AFP levels) should prompt consideration of this disease, leading to targeted testing for HT1. Molecular tests, specifically the identification of FAH gene mutations, are required to confirm an HT1 diagnosis [1,3]. Our patient was found to have the FAH gene mutation 000137.4 ((FAH): c.[1062+5G>A]; [1062+5G>A]), which is the most common mutation in both Northern Europe and Canada.

Experts in this field emphasize that treatment with the drug nitisinone, also known as 2-(2-nitro-4-trifluoromethylbenzoyl) cyclohexane-1,3-dione (abbreviated as NTBC), at a dose of 1 mg/kg/day should be initiated upon suspicion of HT1, and the response to this drug is typically rapid, with clinical improvement occurring within one week [3,10]. A number of studies have demonstrated the undeniable effectiveness of this drug [11,12,14].

In the case of our patient, treatment with nitisinone was initiated upon suspicion of the disease, following a 4-fold increase in tyrosine concentration in the blood and a 4000-fold increase in SA concentration in the urine. We observed a clinical response to this treatment within the first day, leading us to suspend preparations for liver transplantation. According to some researchers, liver transplantation should be considered in cases where there is no response to nitisinone treatment after approximately one week or when there is continuous severe coagulopathy and/or encephalopathy within 2–3 days [3].

In addition to treatment with nitisinone, a diet low in phenylalanine/tyrosine is necessary. Since this medication increases the concentration of tyrosine in the blood, control of phenylalanine and tyrosine intake in the diet must begin immediately after the diagnosis is established and must be consistently maintained to prevent the formation of tyrosine crystals in the cornea, which could lead to corneal ulcerations [10,15,16]. Experts in this field recommend restricting the amount of tyrosine in the diet to maintain a plasma tyrosine concentration of 200–600 μmol/L [3,10]. It is unclear whether there is a correlation between lower tyrosine levels and the absence of clinical complications in HT1 [17]. However, dietary restrictions are essential to avoid complications, such as corneal damage, which is now recognized as associated with increased tyrosine levels in blood plasma [1,2,3,15]. In our patient’s case, foods rich in phenylalanine/tyrosine were restricted from the diet and after a few months of treatment, a special medical protein product without any tyrosine or phenylalanine was additionally prescribed.

When treating a patient with NTBC throughout their life, it is recommended to continuously monitor liver enzymes (ALT, AST, GGT, ALP), total bilirubin and its fractions, and liver function indicators (SPA, albumin). According to a study conducted in Finland, the liver function of 13 out of 14 patients treated with nitisinone normalized within 31 months [11]. The elevated AFP level should gradually decrease in the first years of treatment [3]. In our patient’s case, the AFP level gradually decreased, but the liver enzyme GGT began to increase after 1 month of treatment. Prescribing ursodeoxycholic acid—although we did not find any data in the literature about the use of this drug in HT1—yielded a positive effect.

Regular AFP measurements are of utmost importance when monitoring patients with HT1, as it is a useful indicator of the development of hepatocellular carcinoma. If AFP does not decrease consistently or increases, imaging studies (abdominal computed tomography (CT) or abdominal magnetic resonance imaging (MRI)) should be performed promptly. However, consistently normal AFP values do not guarantee the absence of HCC [3].

Long-term outcomes in patients with HT1, especially the emergence of HCC, kidney disease, ophthalmologic complications, and the onset of intellectual delays, are unknown, making continuous care essential. Continuous care is crucial, involving periodic measurements of AFP levels in the blood and imaging studies (abdominal ultrasound (US), CT, or MRI)—initially every 3–6 months and later reduced to once a year [3,17,18], or immediately if AFP increases [3]. Patients with HT1 are at a significantly high risk of developing severe liver disease and hepatocellular carcinoma. This risk can be reduced to less than 1% if NTBC treatment is initiated in early infancy and continued without interruption [3]. Lifelong monitoring for these liver complications in HT1 patients is of utmost importance. If treatment with nitisinone and a low-tyrosine diet is effective, long-term survival can be expected [3,14]. According to data from Sniderman King L. et al., a combination treatment of nitisinone and a low-tyrosine diet resulted in over 90% survival, normal growth, improved liver function, prevention of cirrhosis, correction of renal tubular acidosis, and improvement of secondary rickets [10].

Scientific studies indicate that better results are achieved when nitisinone is administered alongside a specialized diet, with cooperation from patients and their parents, consistent medication and diet adherence, and regular visits to their physicians [12]. Therefore, in the case described in our article, efforts have been made to ensure constant communication between physicians and the patient’s parents.

## 4. Conclusions

It is crucial to diagnose HT1 as early as possible to reduce or fundamentally eliminate complications associated with the disease, including progressive liver failure, kidney dysfunction, hypophosphatemic rickets, dangerous neurological crises, and early development of hepatocellular carcinoma (HCC). This can be achieved only through the implementation of universal newborn screening for tyrosinemia and the initiation of nitisinone (NTBC) treatment before the age of 1 month. However, in countries where such screening is not conducted, physicians must be aware of and consider this extremely rare disease. They should be vigilant, paying attention to even minimal changes in laboratory test results, such as unexplained anemia, accompanied by thrombocytopenia and neutropenia, and conduct more detailed examinations to determine the cause of these variations.

The earlier the diagnosis is made and the more prompt the initiation of treatment with nitisinone together with a proper diet, the better the outcomes of the patients tend to be. Nitisinone does not cure the disease but only reduces disease progression and the development of associated complications. It is evident that universal newborn screening for tyrosinemia, timely administration of nitisinone, as well as appropriate and consistent patient monitoring, can prevent the progression of the disease and improve the quality of life for patients. Due to the very high risk of developing severe liver disease and hepatocellular carcinoma, continuous monitoring of these patients throughout their lives is absolutely crucial.

## Figures and Tables

**Table 1:** Results of laboratory investigations of the patient. (Empty cells indicate that the test was not performed at the given time. Initial time stamps indicate the time after the patient was admitted into the hospital, with Day 1 being the day of admission).

Laboratory Test	Reference Range	Results of the Laboratory Test						
		Day 1	Day 5	After Commencing Nitisinone Treatment				
				2 Days	30 Days	2 Months	5 Months	9 Months
Complete blood count								
Erythrocytes (×10^12^/L)	3.7–6	4.3	2.92	3.53	3.67	3.98	4.44	4.18
Hemoglobin (g/L)	105–155	103	73	95	101	108	120	121
Leukocytes (×10^9^/L)	6–11	12.2	8.4	7.1	5.6	6.0	9.2	5.9
Neutrophils (×10^9^/L)	1.3–7.2	2.1	1.9	1.6	1.4	1.6	3.6	2.9
Lymphocytes (×10^9^/L)	1.5–8.1	9	5.3	4.7	3.6	3.7	4.2	2.2
Platelet count (×10^9^/L)	150–450	204	41	130	142	155	242	249
CRP (mg/L)	0–5	5	59.3	25.6				
Liver function test								
Total bilirubin (µmol/L)	1.7–15.4	22.16	40.31	47.54	22.55	16.46	14.2	11.32
Conjugated bilirubin (µmol/L)	0–3.4	7.45	21.35	22.43	8.13	5.13	3.7	3.62
ALT (IU/L)	7–45	29	46	26	35	44	32	30
AST (IU/L)	8–50	74	207	77	82	90	65	51
ALP (IU/L)	108–317	1056	741	700	493	448	324	336
GGT (IU/L)	3–22	227	236	189	319	277	99	81
SPA (%)	70–130	25	14	20	40	47	59	66
INR	0.9–1.2	2.2	3.2	2.4	1.6	1.4	1.3	1.2
Albumin (g/L)	35–50	34.7	26	26.3	34.3	37.1	37.5	40.1
Ammonia (µmol/L)	16–53	119	101	68				
AFP (kU/L)	0.5–9.17		47,863.6		5797.7	2029.7	362.4	100.2

CRP—C-reactive protein; ALT—alanine transaminase; AST—aspartate transaminase; ALP—alkaline phosphatase; GGT—gamma-glutamyltransferase; SPA—Owren’s Stago Prothrombin Assay; INR—international normalized ratio; AFP—α-fetoprotein.

## Data Availability

The original contributions presented in the study are included in the article, further inquiries can be directed to the corresponding author.
